# Nebivolol Polymeric Nanoparticles-Loaded In Situ Gel for Effective Treatment of Glaucoma: Optimization, Physicochemical Characterization, and Pharmacokinetic and Pharmacodynamic Evaluation

**DOI:** 10.3390/nano14161347

**Published:** 2024-08-14

**Authors:** Pradeep Singh Rawat, Punna Rao Ravi, Mohammed Shareef Khan, Radhika Rajiv Mahajan, Łukasz Szeleszczuk

**Affiliations:** 1 Department of Pharmacy, Birla Institute of Technology and Science, Pilani Hyderabad Campus, Jawahar Nagar, Kapra Mandal, Medchal District, Hyderabad 500078, Telangana, India; p20170300@hyderabad.bits-pilani.ac.in (P.S.R.); p20190064@hyderabad.bits-pilani.ac.in (M.S.K.); p20200469@hyderabad.bits-pilani.ac.in (R.R.M.); 2 Department of Organic and Physical Chemistry, Faculty of Pharmacy, Medical University of Warsaw, Banacha 1 Str., 02-093 Warsaw, Poland; lukasz.szeleszczuk@wum.edu.pl

**Keywords:** nebivolol hydrochloride, glaucoma, in situ gel, polycaprolactone nanoparticles, optimization, physical characterization, ocular pharmacokinetics

## Abstract

Nebivolol hydrochloride (NEB), a 3rd-generation beta-blocker, was recently explored in managing open-angle glaucoma due to its mechanism of action involving nitric oxide release for the vasodilation. To overcome the issue of low ocular bioavailability and the systemic side effects associated with conventional ocular formulation (aqueous suspension), we designed and optimized polycaprolactone polymeric nanoparticles (NEB-PNPs) by applying design of experiments (DoE). The particle size and drug loading of the optimized NEB-PNPs were 270.9 ± 6.3 nm and 28.8 ± 2.4%, respectively. The optimized NEB-PNPs were suspended in a dual-sensitive in situ gel prepared using a mixture of P407 + P188 (as a thermo-sensitive polymer) and κCRG (as an ion-sensitive polymer), reported previously by our group. The NEB-PNPs-loaded in situ gel (NEB-PNPs-ISG) formulation was characterized for its rheological behavior, physical and chemical stability, in vitro drug release, and in vivo efficacy. The NEB-PNPs-loaded in situ gel, in ocular pharmacokinetic studies, achieved higher aqueous humor exposure (AUC_0–t_ = 329.2 ng × h/mL) and for longer duration (mean residence time = 9.7 h) than compared to the aqueous suspension of plain NEB (AUC_0–t_ = 189 ng × h/mL and mean residence time = 6.1 h) reported from our previous work. The pharmacokinetic performance of NEB-PNPs-loaded in situ gel translated into a pharmacodynamic response with 5-fold increase in the overall percent reduction in intraocular pressure by the formulation compared to the aqueous suspension of plain NEB reported from our previous work. Further, the mean response time of NEB-PNPs-loaded in situ gel (12.4 ± 0.6 h) was three times higher than aqueous suspension of plain NEB (4.06 ± 0.3 h).

## 1. Introduction

Beta-blockers from the first-generation class are the choice of treatment for glaucoma. However, limited precorneal retention time, rapid nasolacrimal drainage, and absorption into systemic circulation lead to lower ocular bioavailability and increased systemic safety concerns with the currently available treatments [1]. To overcome these challenges, a new third-generation beta adrenolytic drug, nebivolol (NEB), with its unique nitric oxide release mechanism via the L-arginine/NO/cGMP pathway (that modulates aqueous humor flow from trabecular meshwork and has a potential neuroprotective effect) was incorporated into novel drug delivery systems [2]. The main reason for poor ocular bioavailability is due to anatomical and physiological aspects of the eye that act as barriers to drug absorption and penetration in reaching the target site. In our previous published work, we designed NEB-loaded dual-sensitive in situ gel that significantly helped increase residence time, which led to improved pharmacokinetic and pharmacodynamic effects of NEB suspension-loaded in situ gel over conventional NEB suspension [3].

To further improve the effectiveness of ocular delivery and efficacy of NEB, we explored the idea of a nanotechnology-based drug delivery system that protects the drug from metabolic degradation or efflux transporters by limiting the availability of the free drug. Further, the nanoparticles can be taken up by direct pathways though the cornea and reach the aqueous humor, in which the drug can be released in a sustained manner for a longer duration [4]. Recently, researchers have explored the approach of loading polymeric nanocarrier formulations on in situ gels for improving the efficiency of drug delivery to the intra-ocular tissues and addressing issues such as tear fluid dilution, nasolacrimal drainage, and low pre-corneal residence time of conventional formulation while also providing dose accuracy and patient convenience in treatment of glaucoma [5,6]. Polymeric nanocarriers can load both hydrophilic and lipophilic drugs with higher drug loading, offer better physical stability and controlled drug release, and are amenable to surface modification. Drug-loaded nanoparticles with a particle size range of 50 to 400 nm are optimal for ocular administration in terms of their capacity to cross ocular barriers and enter intra-ocular tissues without causing ocular discomfort [7].

Poly(caprolactone) (PCL) is a synthetic polymer of ε-caprolactone monomers formed via the induction of ring-opening polymerization reaction. PCL undergoes biodegradation due to the hydrolysis of the ester bond via carboxyl esterase enzymes to form carboxylic acid [8]. Ala H. Salama et al.’s research on nanoparticles encapsulated ofloxacin ocular in situ gel revealed higher corneal absorption and improved antibacterial activity in contrast to the commercial eye drop product Oflox [9]. Pankaj et al. developed brimonidine tartrate-encapsulated vitamin E-tocopheryl polyethene glycol succinate (TPGS)-PCL nanoparticles that showed enhanced penetration, prolonged drug release for over 24 h, and a higher decrease in IOP than conventional formulation in glaucoma-induced rabbit model [10].

In our current research, we designed and evaluated polycaprolactone polymeric nanoparticles of NEB (NEB-PNPs) to overcome the problems associated with conventional ocular formulation of NEB suspension. The formulation components and process parameters were optimized by applying DoE. Further, the optimized NEB-PNPs formulations were evaluated for physicochemical characteristics, in vitro drug-release properties, and in vivo pharmacokinetic and pharmacodynamic parameters.

## 2. Materials and Methods

### 2.1. Materials

NEB and deuterated NEB (IS in bioanalysis) were obtained from MSN Laboratories (Hyderabad, India) and Bio-organics Limited (Bangalore, India), respectively. Poloxamer 407 (P407) (average molecular weight: 12,600 Da), kappa-carrageenan (κCRG) (average molecular weight: 788.65 KDa, viscosity of 10–25 mPa·s for 0.3% *w*/*v* aqueous solution at 25 °C), poloxamer 188 (P188) (average molecular weight: 8400 Da), PCL (average molecular weight: 14,000 Da), and polyvinyl alcohol (average molecular weight: 160,000 Da) were sourced from Sigma-Aldrich Private Limited (Mumbai, India). LC-MS-grade acetonitrile and methanol were sourced from Thermo Fischer Scientific (Mumbai, India). N-methyl pyrrolidone (NMP) and trehalose SG were procured from Tokyo Chemical Industries (India) Private Limited, Hyderabad, India, and Hayashibara Company Limited (Okayama, Japan), respectively. Ammonium acetate was purchased from Sisco Research Laboratories (SRL) Private Limited (Mumbai, India). HPLC-grade water obtained from Millipore^®^ Milli-Q system (Burlington, MA, USA) was used in the analysis of samples. Male rabbits (New Zealand white, 2.5 kg and 10 months old) were purchased from Vimta Labs (Hyderabad, India).

### 2.2. Analytical and Bioanalytical Methods

Various aqueous samples, including formulation drug-loading/entrapment studies and in vitro NEB release studies, were analyzed using an HPLC-UV analytical method. The developed method was similar to the method published by Zoltan et al., with minor changes [11]. The proposed detection and quantification method for NEB has a shorter run time per sample, and it is linear, precise, and accurate in the 100–10,000 ng/mL concentration range. A Kinetex^®^ C18 column (250 mm length × 4.6 mm internal diameter × 5 μm particle size) (Phenomenex, Torrance, CA, USA) and a mobile phase consisting of 0.1% (*w*/*v*) orthophosphoric acid in water (pH 2.5 ± 0.05) (as aqueous phase) and acetonitrile (as organic phase) in a 57:43 (*v*/*v*) ratio 0.8 mL/min flow rate were used in the analysis.

A validated LC-MS/MS method in the linear range of 0.43–750 ng/mL, published earlier by our group [12], was utilized to analyze the aqueous humor and plasma concentrations of NEB from the samples obtained from ocular pharmacokinetic studies. A Zorbax SB-C18 reverse-phase column (100 mm length × 4.6 mm internal diameter × 3.5 μm particle size) with a mobile phase comprising of an organic phase (mixture of acetonitrile and methanol in a 30:70 (*v*/*v*) ratio) and an aqueous phase (ammonium acetate buffer (5 mM), pH 3.5 ± 0.05) in a 75:25 (*v*/*v*) ratio at 0.6 mL/min flow rate was used in the analysis. NEB was extracted from plasma and aqueous humor samples by precipitating the proteins.

### 2.3. Selection of Excipients and Process Parameters for the Preparation of NEB-PNPs

NEB-PNPs were prepared by nanoprecipitation technique, involving the solvent–antisolvent method [13]. The effect of various stabilizers (polyvinyl alcohol, polyvinyl pyrrolidone K30, and P407) and manufacturing process parameters (homogenization speed, homogenization time, and stirring time) on the physicochemical characteristics like particle size (PS), zeta potential (ZP), polydispersity index (PDI), drug loading (DL (%)), and efficiency of entrapment (EE (%)) of NEB-PNPs were evaluated.

### 2.4. Formulation of NEB-PNPs

Briefly, 10 mg of NEB and 30 mg of PCL were dissolved in 1.0 mL of NMP in a centrifuge tube to form the organic phase. The aqueous phase was a monophasic solution of the stabilizer PVA (0.75% *w*/*v*, varied within the range as per the design) in water. The organic phase containing NEB + PCL in NMP was added slowly (drop by drop) to the aqueous phase using a syringe (fixed with a needle having 0.72 mm internal diameter) while subjecting to homogenization (Polytron PT 3100D, Kinematica, Malters, Switzerland) to form a nanosuspension. The resultant nanosuspension was then stirred on a magnetic stirrer at 500 rpm for 1 h at 25 ± 0.1 °C (room temperature) to allow its equilibration for stable PS and PSD. The resultant NEB-PNPs were transferred to 50 mL tubes and centrifuged at 9486× *g* (40 min, 10 °C) to form a pellet. The resultant pellet (after washing twice with Milli-Q water) was redispersed in 5 mL of 5% *w*/*v* trehalose solution (prepared using Milli-Q water as the solvent) and frozen for 8 h at −80 °C before subjection to the lyophilization process. The lyophilized NEB-PNPs were stored at refrigerated conditions (2–8 °C). An aqueous suspension of NEB-PNPs (NEB-PNPs-Susp) was formed by dispersing the lyophilized powder of NEB-PNPs in deionized water. NEB-PNPs-Susp was used in various studies, including the physico-chemical characterization, in vitro drug release, and in vivo efficacy studies.

#### 2.4.1. Optimization by Applying Design of Experiments (DoE) for the Preparation of NEB-PNPs

In the preliminary formulation trials, three independent factors (X_1_—amount of PCL; X_2_—concentration of stabilizer, and X_3_—homogenization speed) were found to affect two critical responses, namely the PS (Y_1_) and DL (%) (Y_2_) of NEB-PNPs. As there were only three independent factors, a direct optimization design was applied to determine the regression equation relating the three independent factors to each critical response (Y_1_ and Y_2_). Prior to the optimization studies, a few more formulation trials were conducted to set the levels (lower level (−1) and upper level (+1)) of the three factors. Box–Behnken design (BBD), which is a quadratic response surface method (RSM), was used for the optimization of the three independent factors. The three independent factors with their three levels (low (−1), medium (0), and high (+1)) and the two critical responses with their constraints used in BBD are shown in Table S1. The Design Expert (DoE) software (version 13, Stat-Ease Inc., Minneapolis, MN, USA) was employed in obtain the experimental runs of the optimization studies.

In the BBD, the three independent factors were studied at three levels to optimize their effect on the two critical responses. The BBD generated 17 experimental runs (with five center-point runs) to assess the main effects, two-factor interaction effects, and quadratic effects. The five center-point runs helped to determine the pure error as well as the precision of the method of preparation of NEB-PNPs. The properties of design used in the optimization of NEB-PNPs are summarized in Table 1.

#### 2.4.2. Optimization and Validation of the Regression Equations

The optimum levels for the three factors to prepare NEB-PNPs with desired PS and DL (%) were determined using a simultaneous optimization technique involving desirability functions. Out of multiple solutions given by the Design Expert software, the solution (with the levels of X_1_, X_2_, and X_3_) with the highest overall desirability was fixed as the optimum to formulate the optimized NEB-PNPs. The solution recommended by the software was validated by preparing three independent replicate formulations of NEB-PNPs using the levels of the factors in the optimum solution and evaluated for their critical responses (Y_1_—PS and Y_2_—DL (%)). The predicted data of the two critical responses were determined by substituting the fixed conditions of the three factors (provided by the desirability function) in the corresponding regression equations of the critical responses. The observed responses of the formulations (prepared in the validation runs) and their corresponding predicted data were compared statistically at the 5% level of significance.

### 2.5. Evaluation of Various Physical Characteristics of NEB-PNPs

#### 2.5.1. Analysis of Particle Size and Zeta Potential of NEB-PNPs

Zetasizer Nano ZS (Malvern Instruments, Worcestershire, U.K.) was used to measure the PS and particle size distribution (in terms of PDI) as well as the ZP of NEB-PNPs. A laser of 633 nm from a helium–neon lamp was used to irradiate the sample. The scattered light from the sample detected at an angle of 173°. The NEB-PNPs nanosuspension (freshly prepared) was diluted with Milli Q water (10× dilution) and placed in the sample compartment, set at 25 °C, for equilibration. After 2 min of equilibration, the sample was analyzed. For each sample, the average of three measurements were recorded to determine the PS, PDI, and ZP values.

#### 2.5.2. Analysis of Drug Loading and Entrapment Efficiency of NEB-PNPs

The DL (%) and EE (%) of NEB PNPs were determined using direct and indirect methods. For the direct method, the pellets obtained from the centrifugation of freshly prepared NEB-PNPs were washed twice with 5 mL of Milli-Q water and vacuum-dried. The weighed quantity of the dried pellet was dissolved in 1.0 mL NMP by vortexing the sample for 5 min, followed by its dilution in Milli-Q water and analysis using the HPLC-UV method described in Section 2.2. In the indirect method, the supernatant obtained after centrifugation of freshly prepared NEB-PNPs was collected and diluted to determine the amount of NEB present in the continuous phase of the nanosuspension. The DL (%) and EE (%) of NEB-PNPs in the direct method were determined using appropriate formulae [14,15].

### 2.6. Scanning Electron Microscopy (SEM) Imaging of the Optimized NEB-PNPs

An FE-SEM (FEI, Apreo LoVac, ThermoFisher Scientific, Waltham, MA, USA) was used to image the optimized NEB-PNPs technique [16] to study their shape and surface properties. A small sample (40 μL) of the NEB-PNPs nanosuspension was placed on an aluminum stub and dried for 12 h at room temperature (25 °C). In the presence of argon gas, gold was coated on the surface of sample using a Leica EM ACE200 sputter-coater (Wetzlar, Germany). Finally, the sample (coated with gold) was imaged at an acceleration voltage of 5 kV.

### 2.7. Differential Scanning Calorimetry (DSC) of the Optimized NEB-PNPs

Thermal analysis for NEB, PCL, powder mixture of NEB with all the formulation excipients used in the preparation of NEB-PNPs, and lyophilized NEB-PNPs was performed using Shimadzu DSC-60 (TA-60 WS, Kyoto, Japan) [17]. Aluminum pans were filled with the samples (2–3 mg) and crimp-sealed. The sample pan and an empty aluminum pan (reference) were placed in the DSC chamber set at 25 °C. Both the pans were equilibrated for 2 min before recording the thermograms at a heating rate of 10 °C/min, in a temperature range between 25 to 250 °C. Nitrogen was purged at a flow rate of 50 mL/min into the DSC chamber during the analysis.

### 2.8. X-ray Diffractometric Analysis of the Optimized NEB-PNPs

The NEB-PNPs were subjected to X-ray diffractometric analysis to determine the physical nature of NEB entrapped in the nanoparticles [18]. A Rigaku X-ray diffractometer (Ultima IV, The Woodlands, TX, USA) with copper anode (1.54 Å) set at 60 kV and 60 mA was used in the analysis. The X-ray diffractograms of NEB, PCL, a powder mixture of NEB with all the formulation excipients used in the preparation of NEB-PNPs, trehalose (cryoprotectant used in the lyophilization process), and freeze-dried NEB-PNPs were captured in the 2-theta range of 5–50° and at a scanning rate of 4 degrees/min.

### 2.9. Preparation of NEB-PNPs-Loaded Dual-Sensitive In Situ Gel

In an earlier research work, we reported the optimization of NEB-loaded dual-sensitive in situ gel (ISG) containing a combination of P407 + P188 (thermo-sensitive polymers) and κCRG (ion-sensitive polymer) [3]. In the current work, lyophilized powder of the optimized NEB-PNPs (875 mg) was loaded onto the dual-sensitive, blank in situ gel (ISG) (1 mL) to form NEB-PNPs-loaded dual-sensitive in situ gel (NEB-PNPs-ISG). The nanoparticles were added to the ISG by continuous agitation at 500 rpm on a magnetic stirrer for 30 min while maintaining the temperature at 25 °C.

### 2.10. Rheological Evaluation of NEB-PNPs-ISG Formulation

An Anton Paar MCR 302 rheometer (Graz, Austria) was used for rheological evaluation of NEB-PNPs-ISG and blank ISG. Parallel-plate geometric method was used to study all the samples. For each sample, the linear viscoelastic region (LVER) was identified by conducting experiments involving both frequency and amplitude sweep. Then, rheological properties were analyzed in the temperature range of 25 °C to 40 °C using the oscillatory mode. The gelation property of blank ISG and NEB-PNPs-ISG was assessed as a function of temperature. The evaluation was performed in the presence of freshly prepared STF (pH 7.4 ± 0.05). The changes in both loss tangent (tan δ) as well as storage modulus (G′) as a function of temperature were recorded for all the samples. The in situ gelling properties of NEB-PNPs-ISG and blank ISG, in different conditions, were compared based on their tan δ and G′ values and the corresponding plots [19,20].

### 2.11. In Vitro Drug-Release Studies of NEB-PNPs-Susp and NEB-PNPs-ISG Formulations

Dialysis membrane method was employed to study the release of NEB from NEB-PNPs-Susp and NEB-PNPs-ISG in the in vitro conditions [21]. First, 40 µL of test formulation (equivalent to 0.12 mg of NEB) was placed in a dialysis bag (MWCO: 3500 Da) and sealed from both ends and suspended in a beaker containing 100 mL of simulated tear fluid (STF, pH 7.4 ± 0.05) with Tween 80 (0.5% *w*/*v*) as the dissolution media. The temperature was set at 34 ± 0.5 °C, while the stirring rate was fixed at 75 rpm during the dissolution study. Samples (2 mL) were drawn at 0.5 h, 1 h, 2 h, 4 h, 6 h, 8 h, 12 h, 16 h, 18 h, and 24 h from the dissolution media. Fresh media (pre-heated to 34 ± 0.5 °C) of equal volume (2 mL) were replaced at each sampling point. The samples were centrifuged at 10,000 rpm, and the clear supernatant was separated. The supernatant was analyzed, after suitable dilutions, for quantification of NEB using the fit-for-purpose HPLC-UV method, as mentioned in Section 2.2. The cumulative percentage drug-release data of both formulations was fitted into empirical models using Phoenix^®^ WinNonlin software (version 8.3.5.340, Certara Inc., Durham, NC, USA) [22,23].

### 2.12. In Vivo Evaluation of Optimized NEB-PNPs and NEB-PNPs-ISG Formulations

#### 2.12.1. Ocular Pharmacokinetic Studies

New Zealand white rabbits, weighing approximately 2.5 kg (with clinically normal eyes), were used to compare the pharmacokinetic performance of the optimized NEB-PNPs-Susp and NEB-PNPs-ISG administered through ocular route. The animal ethics committee of Vimta Labs (Hyderabad, India) examined and approved the protocol for all the in vivo studies (Protocol No.: VLL/1122/NG/1099R). The rabbits were acclimated (22 ± 1 °C; 55 ± 10% RH and 12 h light–dark cycle) for one week before administering the formulations. In each treatment group, the test formulation (40 μL, equivalent to 0.125 mg of NEB) was instilled into the lower cul-de-sac of both the eyes of each rabbit using a calibrated micropipette. The drug dose was maintained the same at 0.05 mg/kg per eye for both the formulations. Each treatment was administered in two rabbits. Using a sterile hypodermic needle (30 gauge), samples of aqueous humor (70 μL) were collected from the anterior chamber of the eye. The rabbits were anesthetized using isoflurane (2% *v*/*v*) in a chamber just before sample collection, at each sampling point. To collect aqueous humor, a sparse sampling method was followed. The samples were collected pre-dose as well as 0.5 h, 1 h, 2 h, 4 h, 8 h, 12 h, and 24 h after administering the formulations. Pre-dose samples were collected from all the rabbits at least 1 h before the dose administration. The samples were collected from rabbit 1 and rabbit 3 at 0.5 h, 2 h, 8 h, and 24 h, while from rabbit 2 and rabbit 4, the samples were collected at 1 h, 4 h, and 12 h. Each data point in the aqueous humor time course is the mean (±SD) of four samples collected from both the eyes of two different rabbits. The data collected from rabbits 1 and 3 were then pooled with data from rabbits 2 and 4 to construct the entire aqueous humor time course. To determine the systemic exposure of NEB, samples of blood (0.25 mL) were collected pre-dose and at 0.5 h, 1 h, 2 h, 4 h, 8 h, 12 h, and 24 h from the ear vein of all the rabbits (in a serial sampling method). The samples were collected into Eppendorf tubes containing 200 mM K2EDTA (20 μL of K2EDTA per mL of blood) to prevent the coagulation of blood [24]. Blood samples were subjected to centrifugation to obtain plasma. The analytical method described in Section 2.2 was used to analyze the NEB concentration in all the plasma and aqueous humor samples collected in the study. The NEB concentration versus time profiles in plasma and aqueous humor were subjected to non-compartmental analysis to determine the pharmacokinetic parameters [25].

#### 2.12.2. Ocular Pharmacodynamic Studies of NEB-PNP Formulations

The pharmacodynamic efficacy studies of the optimized NEB-PNPs and NEB-PNPs-ISG (NEB at 0.05 mg/kg of rabbit in each eye) were performed in New Zealand white albino rabbits (n = 3 for each treatment group). The percentage reduction in the intra-ocular pressure (ΔIOP (%)) from baseline was determined at different time points after administering the formulations. The baseline IOP was measured in both eyes before administering the formulations using a calibrated TONO-PEN XL tonometer (Reichert, Munich, Germany) [26]. In a treatment group, 40 μL of the formulation was instilled at once into the lower part of the cul-de-sac of both the eyes of the rabbit and closed immediately for 10 s. The IOP values were noted at 0 h (pre-dose), 1 h, 2 h, 4 h, 6 h, 12 h, and 24 h in both the eyes of each rabbit. The ΔIOP (%) values at each time point were determined using Equation (1):

(1) $$∆IOP(\%)=\frac{\left({IOP}_{Pre}-{IOP}_{t}\right)}{{IOP}_{Pre}}\times 100$$

where ${IOP}_{Pre}$ is the intraocular pressure at pre-dose (just before administering the treatment), and, ${IOP}_{t}$ is the intraocular pressure at the time t following the administration of the treatment.

## 3. Results and Discussion

### 3.1. Preliminary Trials for the Preparation of NEB-PNPs

NEB was found to have high solubility and good stability in NMP among the various solvents screened for the preparation of NEB-PNPs. The selection of a stabilizer was another crucial factor in regulating particle size during the preparation of nanoparticles. Among the various stabilizers screened for the preparation of NEB-PNPs, PVA was selected, as it resulted in nanoparticles with smaller PS with low PDI, good physical stability, and relatively high DL (%). The optimum levels of the formulation factors, such as polymer amount, stabilizer concentration, and process conditions such as homogenization speed were optimized using the DoE.

### 3.2. Optimization of NEB-PNPs

The critical responses selected in the optimization design were based on the biopharmaceutical properties, which impact the overall performance of designed nanoparticles. The PS of nanoparticles plays a significant role in the drug release and in the possible direct uptake process of the nanoparticles through the cornea. The PDI is a measure of the efficiency of the manufacturing method in producing nanoparticles of uniform size, and it has been targeted to be below 0.3. The DL (%) of the nanoparticles impacts the amount of nanoparticle powder to be dispersed in the dosing volume for dose administration. Nanoparticles with higher DL (%) can be easily accommodated in small dosing volume (40 µL) for ocular delivery. The EE (%) reflects the manufacturing process efficiency and also the affinity/ability of the polymer (used in the preparation of nanoparticles) to entrap the drug. The ZP plays a critical role in the physical stability of nanoparticles prepared using an electrostatic stabilizer, and values of more than +20 mV or less than −20 mV are desired. However, nanoparticles prepared using steric stabilizers show good physical stability even though their ZP is low.

BBD was used to optimize the factors affecting the critical physico-chemical properties of the NEB-PNPs. BBD is the most suitable quadratic response method to study the effect of factors when their levels are at or close to their extreme levels. The lower level of factor X_2_ (concentration of PVA (% *w*/*v*)) is 0.5% *w*/*v*, which is closer to 0. In the current study, the optimization design matrix using BBD suggested seventeen independent runs (including five center-point runs) with the observed values of PS (Y_1_) and DL (%) (Y_2_) for each of the runs shown in Table S2. The results obtained for PDI (ranged from 0.21 to 0.27), EE (%) (ranged from 96.5% to 96.9%), and ZP (ranged from −4.7 mV to −8.8 mV) in the BBD indicated that there was no significant variation in these properties of the NEB-PNPs across the 17 runs. Narrow and smaller PDI values suggest that the manufacturing method is efficient and reliable in producing NEB-PNPs with uniform PS. The lower ZP of the NEB-PNPs is due to the use of PVA, a non-ionic steric stabilizer that does not impart any charge to stabilize the nanoparticles by forming an electric double layer. Therefore, in the regression analysis of the data obtained from the optimization design using BBD, PS and DL (%) were considered as the critical responses.

#### 3.2.1. Impact of Critical Factors on Particle Size of NEB-PNPs

The PS data of NEB-PNPs manufactured based on the levels of the factors in the seventeen experimental runs in BBD were subjected to regression analysis to determine the mathematical equation relating the PS with the three factors. The second-order polynomial equation to predict the PS (Y_1_) of NEB-PNPs (after excluding the statistically insignificant terms) in the transformed/coded scale is presented in Equation (2) below. Table 2 presents the ANOVA results of the polynomial equation for PS. The regression model for PS was statistically significant (F_cal_ = 36.68 and P_cal_ < 0.01). The lack-of-fit for the regression model was insignificant (F_cal_ = 2.69 and P_cal_ = 0.177), suggesting that the insignificant terms do not affect the values predicted by the regression equation.

(2) $$Y_{1}(nm)=294.99+58.20\;X_{1}-32.61\;X_{3}+26.61\;X_{3}^{2}$$

The *R*^2^_*Adj*_ and *R*^2^_*PRESS*_ values of the polynomial equation for PS were 0.8699 and 0.8006, respectively, with a difference of much less than 0.2 between the two values. Higher *R*^2^_*Adj*_ and *R*^2^_*PRESS*_ values (>0.8) suggest that the PS of NEB-PNPs predicted from the regression equation will be closer to the observed PS. The PS of NEB-PNPs varied, ranging between from 240.6 nm (8th run) and 437.6 nm (11th run) in the optimization design (Table S2).

**Figure 1:**
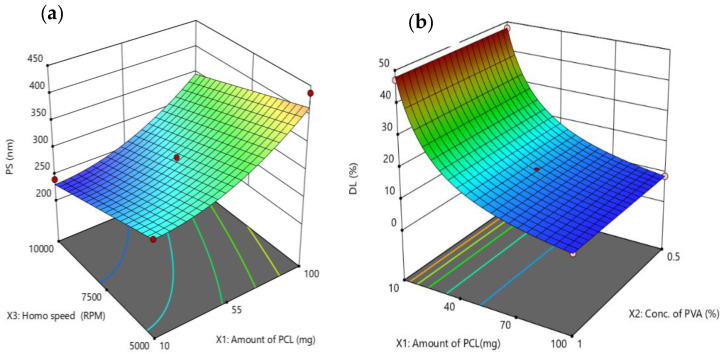
Three dimensional plots demonstrating the impact of significant factors on critical responses: (**a**) PS and (**b**) DL (%) for optimized NEB-PNPs.

The response surface plot for the effect of polymer amount (X_1_) and homogenization speed (X_3_) at a fixed concentration of PVA (X_2_ at 0.75% *w*/*v*) on the PS of NEB-PNPs is presented in Figure 1a. At all levels of homogenization speed, an increase in polymer amount from 10 to 100 mg resulted in an increase in PS of the NEB-PNPs due to the increase in organic phase viscosity, thereby resisting the breakdown of organic-phase droplets during the addition into aqueous phase. Similar observations were made by Sankha et al. when they studied the effect of polymer amount and homogenization speed on the PS of polycaprolactone-based nanoparticles of gefitinib [27].

#### 3.2.2. Impact of Critical Factors on Drug Loading of NEB-PNPs

The DL (%) values obtained from the 17 experimental runs in BBD were subjected to regression analysis to determine the regression equation relating the critical factors with DL (%) of the NEB-PNPs. A simple linear equation, in the transformed scale, for predicting the DL (%) of NEB-PNPs is presented in Equation (3) below:

(3) $$\frac{1}{Y_{2}}\left(\%\right)=0.0679+0.0470X_{1}$$

The ANOVA results of the simple linear equation for DL (%) are presented in Table 2. The DL (%) of NEB-PNPs varied between 8.6% (11th run) and 48% (8th run) in the optimization design (Table S2).

The response surface plot for the effect of polymer amount (X_1_) and concentration of PVA (X_2_) at a fixed homogenization speed (X_3_ at 7500 rpm) on the DL (%) of NEB-PNPs is presented in Figure 1b. At all levels of concentration of PVA, an increase in the polymer amount significantly reduced the DL (%) of the NEB-PNPs, primarily due to an increase in the denominator value (due to an increase in polymer amount) in the equation used for the calculation of DL (%). This is evident from the EE (%) values of the NEB-PNPs, which did not change significantly across the various runs in the optimization design. Therefore, the decrease in DL (%) due to an increase in polymer amount was not due to the decrease in drug entrapment in the NEB-PNPs.

#### 3.2.3. Validation of the Regression Equations for Particle Size and Loading Efficiency

The highest desirability value for simultaneous optimization of PS (with an objective function to minimize, preferably less than 400 nm) and DL (%) (with an objective function to minimize) was found to be 0.9914. The software provided the following optimum levels (in the original scale) for the three critical factors to prepare NEB-PNPs with desired PS and DL (%): X_1_ (amount of polymer) = 25 mg; X_2_ (concentration of PVA) = 0.75% *w*/*v*; X_3_ (homogenization speed) = 10,000 rpm.

The physical characteristics of the optimized NEB-PNPs are presented in Table 3. The statistical comparison between observed data (PS and DL (%)) of the three independent verification runs with the predicted data (determined using the mathematical equations for PS and DL (%)) using Wilcoxon signed-rank test revealed no difference between the values at 5% level of significance.

The NEB-PNPs were manufactured based on the optimum levels of the three critical factors and lyophilized using the procedure discussed in Section 2.4. Further, the lyophilized NEB-PNPs formulation was subjected to different physico-chemical characterization studies.

### 3.3. Physical Characterization of the Optimized NEB-PNPs Using Zeta-Sizer, SEM, DSC, and pXRD

**Figure 2:**
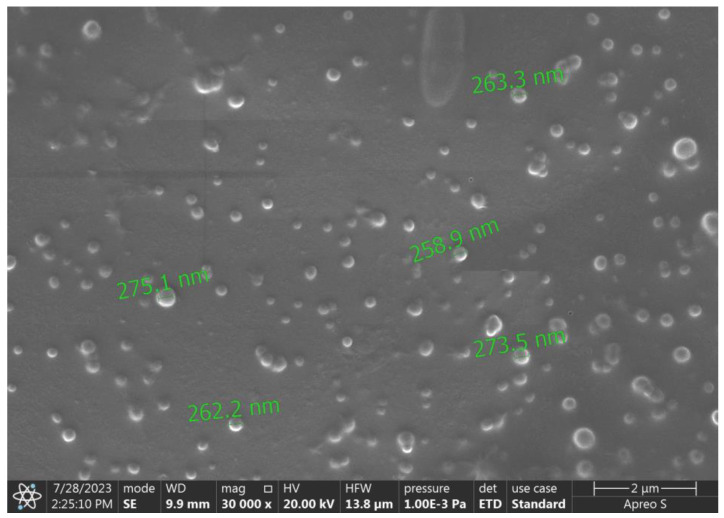
SEM image of the optimized NEB-PNPs.

The optimized NEB-PNPs had an average PS of 276 nm with PDI of 0.105 based on the size analysis using a Zeta-sizer (Figure S1). The lower PDI values (<0.3) reflect the selection of excipients, their proportion, and the process parameters used in the manufacturing to prepare NEB-PNPs with uniform PS. The SEM image of the optimized NEB-PNPs is presented in Figure 2. The optimized NEB-PNPs were spherical in shape, with PS in the range of 263–275 nm, which correlates with the PS measured using Zetasizer. The DSC thermograms of NEB, PCL, the mixture of NEB and the excipients used in the manufacture of NEB-PNPs, freeze-dried NEB-PNPs, and trehalose are presented in Figure S2A. NEB showed an endothermic peak at 228 °C, which corresponds to melting process and the crystalline nature of the drug powder. Similarly, the PCL thermogram showed an endothermic peak at 62 °C, corresponding to the melting process of the crystalline polymer. These endothermic peaks for NEB and PCL in the physical mixture were retained, suggesting that there is no incompatibility between NEB and PCL as well as the other excipients. These distinct peaks at 228 °C (for NEB) and 62 °C (for PCL) were missing in NEB-PNPs, indicating the presence of two substances in the amorphous state in the NEB-PNPs. However, an endothermic peak was observed at 100 °C due to the melting of trehalose used as a cryoprotectant in the lyophilization of NEB-PNPs.

The pXRD diffractograms of various samples analyzed in the study are presented in Figure S2B. Pure NEB showed intense peaks (which do not overlap with trehalose) at 2θ values of 13.13°, 19.20°, and 21.90° (which do not overlap with other excipients), indicating the crystalline nature of the NEB powder. The above peaks, which are specific for the crystalline nature of NEB, were missing in the freeze-dried powder NEB-PNPs, indicating that the NEB is either entrapped in the form of amorphous particles or at a molecular state in the NEB-PNPs. The results from pXRD studies concur with the observations from the DSC analysis.

### 3.4. Rheological Evaluation of NEB-PNPs-ISG Formulation

**Figure 3:**
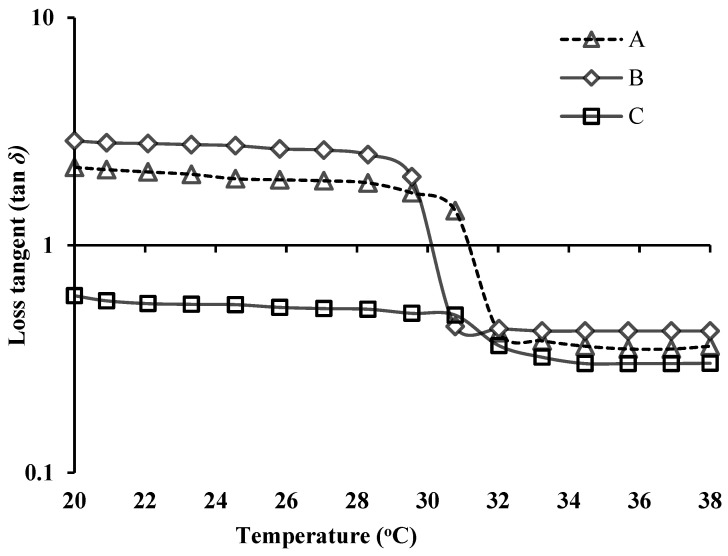
Semi-logarithmic plot of loss tangent versus temperature of the formulations. A—blank ISG; B—NEB-PNPs-ISG; and C—NEB-PNPs-ISG in the presence of STF.

The rheological property of NEB-PNPs-ISG was analyzed by constructing “loss tangent (tanδ) versus temperature” (Figure 3) and “storage modulus (G′, P_a_) versus temperature” (Figure S3) plots, both with and without the presence of STF. The rheological properties of NEB-PNPs-ISG were also compared with blank ISG.

In the experiments involving only the temperature ramp (without STF) for NEB-PNPs-ISG, with the increase in temperature from 28 °C to 33 °C, the tanδ values dropped significantly (31–33 °C), indicating sol-to-gel transition before reaching a plateau above 34 °C. The sudden decrease in tanδ below 1 in 31–33 °C is because of solution-to-gel transformation of the in situ gel due to the thermo-sensitive properties of the mixture of P407 + P188 used in the formulation. This can also be confirmed from the storage modulus (G′, P_a_) versus temperature (without STF), where the G′ showed a sudden increase in the temperatures of 31–33 °C because of the solution-to-gel transformation (Figure S3).

The tanδ values of NEB-PNPs-ISG in the presence of STF were less than 1 even at 20 °C indicating a rapid solution-to-gel transformation due to Na+/K+ ions present in STF. This is due to the ion sensitivity of κCRG used in the formulation. This is also confirmed with much higher values of storage modulus (G′) (>1219.08 Pa) even at 20 °C compared to near 0 values when studied without the STF (Figure S3). Further, NEB-PNPs-ISG exhibited slightly lower tanδ values (accordingly slightly higher storage modulus (G′) values in Figure S3) compared to blank ISG at all temperatures due to the viscosity imparted by the solid content (i.e., NEB-PNPs) dispersed in the in situ gel.

### 3.5. Drug-Release Studies of NEB-PNPs Formulations

**Figure 4:**
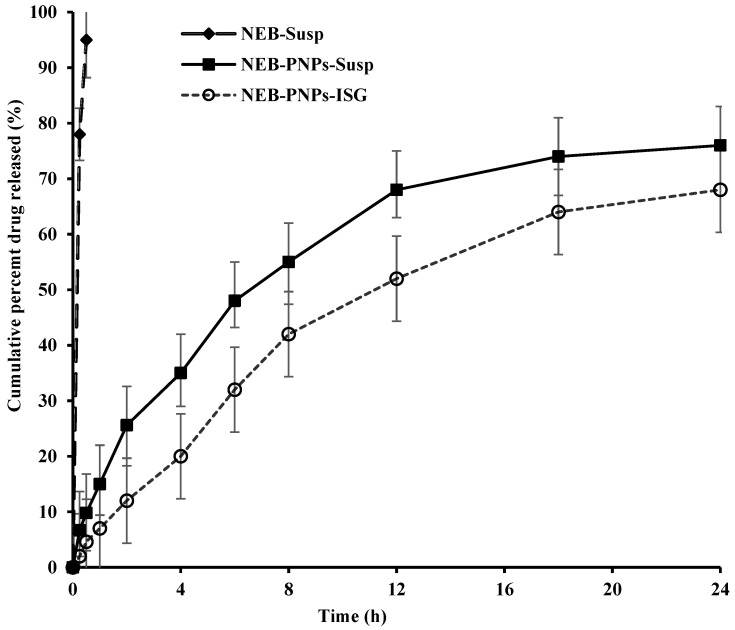
Drug-release profiles of NEB suspension, NEB-PNPs-Susp, and NEB-PNPs-ISG in the in vitro studies. The mean (±SD) of three replicate formulations (n = 3) is presented at each sampling point. Note: Data of NEB-Susp are from our previous published work [3].

NEB has a saturation solubility of 28.62 mg/mL in STF added with Tween 80 (0.5% *w*/*v*). In the drug-release studies, 100 mL of dissolution media containing STF (pH 7.4 ± 0.05) added with Tween 80 (0.5% *w*/*v*) was selected to maintain sink conditions. The cumulative percentages of NEB released vs. time plots of NEB suspension, NEB-PNPs-Susp, and NEB-PNPs-ISG are presented in Figure 4. NEB-Susp showed 100% drug dissolution in 30 min. The drug release from NEB-PNPs-Susp was slow and sustained for more than 24 h, with approximately 73% at the end of 24 h. The drug release from NEB-PNPs-Susp was found to follow the Higuchi kinetic model (R^2^ = 0.966). The analysis the of dissolution data of NEB-PNPs-Susp using Korsmeyer–Peppas suggested an n value of 0.61, indicating that the drug followed non-Fickian diffusion as the primary mechanism of release from the nanoparticles.

The dissolution profile of NEB-PNPs-ISG indicates the drug release was relatively slower and more sustained compared to NEB-PNPs-Susp. The drug release at 2 h was 12% in the case of NEB-PNPs-ISG compared to 25.6% in the case of NEB-PNPs. At 12 h, the drug release was 52% in the case of NEB-PNPs-ISG compared to 68% in the case of NEB-PNPs-Susp. At the end of 24 h of dissolution study, the drug release was approximately 68% from NEB-PNPs-ISG. The release of drug from NEB-PNPs-ISG follows a two-step process, where the drug first releases from the NEB-PNPs-Susp by mechanism of diffusion into the gel matrix, followed by the diffusion of the drug through the gel matrix into the bulk of media or erosion of the gel matrix along with drug release into the dissolution media. The dissolution data of NEB-PNPs-ISG were not modeled using any model-dependent methods due to the limitations of applying such kinetic models to the complex drug-release process from NEB-PNPs-ISG. However, modeling the dissolution data of NEB-PNPs-ISG using empirical models (like Hill model/Weibull model/double Weibull model) indicated that the drug follows the Weibull model (R^2^ = 0.99).

### 3.6. Stability Studies of PNPs Formulations

Figure S4 presents the PS, PDI, and ZP values of lyophilized powder of NEB-PNPs (stored at 25 ± 2 °C and 60 ± 5% RH) and NEB-PNPs-ISG (stored at 2–8 °C) for the freshly prepared formulations (t = 0) and samples collected at different time points over the 60-day study period, respectively. The %RSD values for DL (%) and EE (%) of the freshly prepared formulations and the samples analyzed at different time points during the study period for both formulations were not more than 5%. This suggests that there is no loss or leaching of the drug out of the nanoparticles during the storage conditions.

### 3.7. In Vivo Studies of the NEB-PNPs Formulations

#### 3.7.1. Ocular Pharmacokinetic Studies of NEB-PNPs Formulations

**Figure 5:**
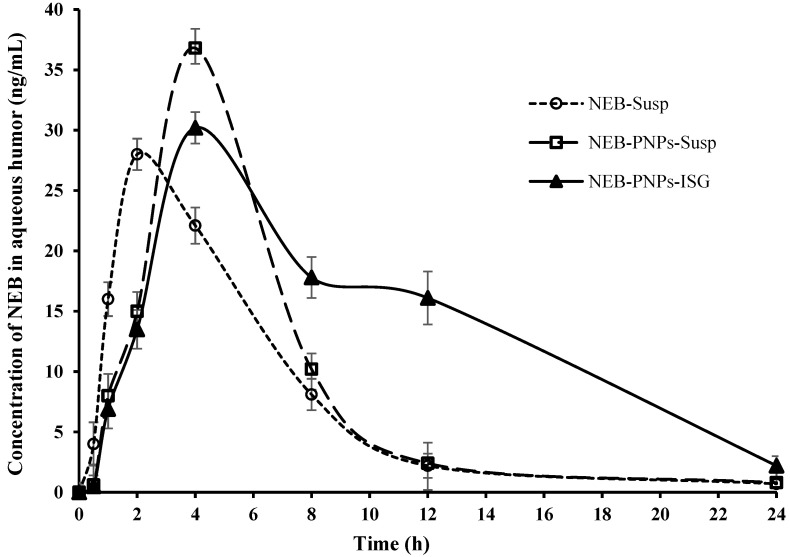
Mean concentration of NEB versus time profiles constructed from the ocular administration of NEB-PNPs-Susp, NEB-PNPs-ISG, and NEB-Susp in aqueous humor. Note: Data of NEB-Susp are reproduced from our previous reported work [3].

Comparative mean concentration versus time profiles of NEB-PNPs-Susp, NEB-PNPs-ISG, and NEB-Susp (for comparison, data are reproduced from our previous published work [3]) in aqueous humor (therapeutic efficacy) and plasma exposure (systemic safety) are presented in Figure 5 and Figure S5, respectively. The ocular pharmacokinetic parameters are discussed in Table 4.

Figure 5 shows that NEB concentrations (C_max_) in the aqueous humor for NEB-PNPs-Susp (36.8 ± 3.2 ng/mL) was significantly higher (P_cal_ < 0.05) than for NEB-PNPs-ISG (30.2 ± 2.1 ng/mL) and NEB-Susp (28.2 ± 3.1 ng/mL). No statistically significant (P_cal_ > 0.05) difference was observed between the C_max_ values of NEB-PNPs-ISG and NEB-Susp. The higher C_max_ of NEB-PNPs-Susp and NEB-PNPs-ISG compared to NEB-Susp could be due to the direct uptake process of the nanoparticles by the cornea and reduction in loss of drug from the precorneal area due to nasolacrimal drainage system. The extent of absorption into the aqueous humor (AUC_0–t_ value) for NEB-PNPs-ISG (329.2 ng × h/mL) was significantly higher than that for NEB-PNPs-Susp (204.4 ng × h/mL) than NEB-Susp (189 ng × h/mL). The MRT_0–∞_ of NEB in the aqueous humor was higher for NEB-PNPs-ISG (MRT_0–∞_ = 9.7 h) compared to both NEB-PNPs-Susp (MRT_0–∞_ = 6.4 h) and NEB-Susp ((MRT_0–∞_ = 6.1 h). The concentration of NEB in aqueous humor was higher and more sustained for NEB-PNPs-ISG due to the higher residence time provided by the in situ gel at the precorneal area than compared to NEB-PNPs-Susp and NEB-Susp, due to its resistance of the tear fluid dilution and clearance of the formulation from the precorneal area.

Figure S5 shows plasma time-course profiles indicating that the C_max_ of NEB-PNPs-ISG (0.58 ± 0.03 ng/mL) was significantly lesser than that of NEB-PNPs-Susp (1.15 ± 0.08 ng/mL) (P_cal_ < 0.05) and NEB-Susp (1.86 ± 0.1 ng/mL) (P_cal_ < 0.01). In addition, the AUC_0–t_ value in plasma (representing the systemic exposure of NEB in plasma) of NEB-PNPs-ISG (8.38 ± 0.56 ng × h/mL) was significantly lesser than that of both NEB-PNPs-Susp (12.1 ± 0.9 ng × h/mL) (P_cal_ < 0.05) and NEB-Susp (20.2 ± 2.7 ng × h/mL) (P_cal_ < 0.01). The MRT_0–∞_ in plasma for NEB-PNPs-ISG (4.6 ± 0.4 h) was also less compared to that for NEB-PNPs-Susp (10.4 ± 1.1) (P_cal_ < 0.05) and NEB-Susp (25.8 ± 1.5 h) (P_cal_ < 0.01). These results suggest that NEB-PNPs-ISG shows significantly lesser systemic exposure and for a shorter duration compared to NEB-PNPs-Susp and NEB-Susp.

Based on higher and sustained aqueous humor-exposure data, accompanied by minimal systemic-exposure data, we can infer that NEB-PNPs-ISG can reduce the dosing frequency of NEB through the ocular route in the treatment of glaucoma.

#### 3.7.2. Ocular Pharmacodynamic Studies

**Figure 6:**
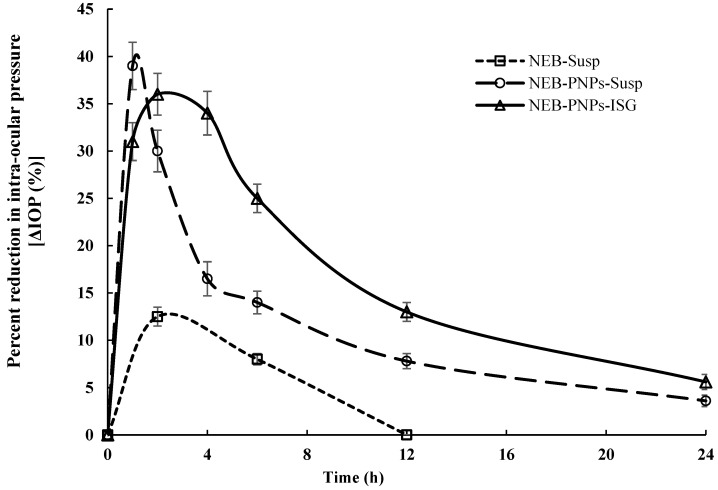
Percent reduction in intra-ocular pressure (ΔIOP (%)) versus time plot of NEB-PNPs-Susp and NEB-PNPs-ISG administered through ocular route in rabbits (n = 6). Note: NEB-Susp profile is reproduced from our previous reported work [3].

A comparative ∆IOP(%) versus time plot of NEB-PNPs-ISG and NEB-PNPs-Susp is shown in Figure 6. Non-compartmental analysis was performed for the area under the curve between t = 0 and t = 24 h (AUC_0–24h_) and mean response time between t = 0 and t = 24 h (MRT_0–24h_). The AUC_0–24h_ of NEB-PNPs-ISG (403.2 ± 16.5% × h) was 1.5-fold higher (P_cal_ < 0.05) compared to NEB-PNPs-Susp (266.2 ± 10.5% × h) and 5.4-fold higher (P_cal_ < 0.01) compared to NEB-Susp (74.2 ± 3.2% × h). No significant difference (P_cal_ > 0.05) was observed in the maximum reduction in the IOP of NEB-PNPs-ISG (peak ∆IOP(%) of 39.2 ± 3.5%) compared to that of NEB-PNPs-Susp (peak ∆IOP(%) of 36.4 ± 2.8%). The mean response time of NEB-PNPs-ISG (MRT_0–24h_ = 12.4 ± 0.6 h) was significantly higher compared to that of NEB-PNPs-Susp (7.8 ± 0.4 h) and NEB-Susp (4.06 ± 0.3 h). These results clearly suggest that the overall pharmacodynamic effect of NEB-PNPs-ISG was much higher and showed a sustained release for a longer duration compared to NEB-PNPs-Susp.

## 4. Conclusions

In this research article, NEB-PNPs were prepared by nanoprecipitation technique, involving the solvent–antisolvent method. An optimization design using DoE was employed to evaluate the effect of critical factors on critical responses like PS and DL (%) of the NEB-PNPs. The NEB-PNPs prepared using the optimal solution provided by DoE were then loaded into a dual-sensitive ISG. In vivo studies were performed for the aqueous suspension of NEB-PNPs (NEB-PNPs-Susp) and NEB-PNPs-loaded ISG (NEB-PNPs-ISG). The pharmacokinetic performance of the NEB-PNPs-ISG (in terms of higher aqueous humor exposure and higher residence time in the aqueous humor) compared to that of NEB-PNPs-Susp and NEB-Susp also reflected its pharmacodynamic performance (overall reduction in the IOP as well as the duration of effect). From the current research work, we can conclude that NEB in a nanocarrier system can be administered as a drop that improves therapeutic outcomes, both in terms of efficacy and safety, compared to the conventional formulation of NEB (NEB-Susp) for patients suffering from glaucoma.

## Figures and Tables

**Table 1:** Summary of properties of the design used in the optimization of NEB-PNPs.

Design used for optimization	Box–Behnken design (BBD): A response surface methodology suitable to optimize and determine the mathematical equations relating three or four independent factors and one or more critical responses.
Criteria for selection the design	Though BBD and central composite design (CCD) are the two most popular optimization designs available, BBD was selected for the following reasons: For the given number of critical factors, fewer runs are required for optimization. Therefore, BBD requires a lesser amount of resources and time compared to CCD for optimization (particularly when quadratic and cubic terms are not significant); BBD avoids runs with extreme levels for all the factors; BBD does not use levels of factors beyond −1 (low) and +1 (high) that are already set from the preliminary trials.
No. of experimental runs used in the design	In the current work, which involves three critical factors, a total 17 runs were performed for optimization (12 factorial runs + 5 center-point runs)
Critical factors	X_1_—amount of PCL; X_2_—concentration of stabilizer; X_3_—homogenization speed
Critical responses	Y_1_—particle size; Y_2_—drug loading (%)
Method to determine optimal solution	Simultaneous optimization of both the critical responses based on the highest overall desirability value.

**Table 2:** ANOVA results of the mathematical equations for PS and DL (%) with the significant terms in the optimization of NEB-PNPs.

Source	Particle Size (Y_1_)				Drug Loading (Y_2_)			
	SS	DF	F_cal_	P_cal_	SS	DF	F_cal_	P_cal_
Model	38,605.75	3	36.68	<0.0001	0.018	1	37,300	<0.0001
X_1_	27,097.92	1	77.23	<0.0001	0.018	1	37,300	<0.0001
X_3_	8508.6	1	24.25	0.0003				
X_3_^2^	2999.23	1	8.55	0.0119				
Residual	4561.33	13			0.00001	15		
Lack-of-Fit	3913.55	9	2.69	0.1773	0.0001	11	1.16	0.482
Pure Error	647.78	4			0.000002	4		
Total	43,167.08	16			0.0177	16		

**Table 3:** The composition and physical characterization data NEB-PNPs manufactured based on the optimum solution given by DoE.

Composition of Optimized NEB-PNPs	* Physicochemical Characteristics of Optimized NEB-PNPs				
	PS (nm)	PDI	ZP (mV)	EE (%)	DL (%)
Organic Phase: 10 mg of NEB + 25 mg of PCL dissolved in 1 mL of NMP Aqueous Phase: PVA (0.75% *w*/*v* in water)	270.9 ± 6.3	0.24 ± 0.03	−8.2 ± 1.2	96.7 ± 0.3	28.8 ± 2.4

NEB—nebivolol; PCL—polycaprolactone; PVA—polyvinyl alcohol; NMP—N-methyl pyrrolidone. * All measurements are expressed as mean ± SD of three replicates.

**Table 4:** NEB pharmacokinetic parameters in the aqueous humor and plasma following the administration of NEB-PNPs-Susp and NEB-PNPs-ISG through ocular route in rabbits.

Biological Matrix	PK Parameters	Units	Treatments		
			NEB-Susp ^#^	NEB-PNPs-Susp	NEB-PNPs-ISG
Aqueous humor	C_max_^a^	ng/mL	28.2 ± 3.1	36.8 ± 3.2	30.2 ± 2.1
	T_max_^b^	h	2.0	4.0	4.0
	AUC_0–t_^c^	ng × h/mL	189	204.4	329.2
	MRT_0–∞_^c^	h	6.1	6.4	9.7
Plasma ^d^	C_max_	ng/mL	1.86 ± 0.1	1.15 ± 0.08	0.58 ± 0.03
	T_max_	h	1.0	2.0	4.0
	AUC_0–t_	ng × h/mL	20.2 ± 2.7	12.1 ± 0.9	8.38 ± 0.56
	MRT_0–∞_	h	25.8 ± 1.5	10.4 ± 1.1	4.6 ± 0.4

^a^ C_max_ is presented as mean ± SD of four measurements. ^b^ T_max_ is presented as median of four measurements. ^c^ The values of AUC_0–t_ and MRT_0–∞_ were obtained by pooling the data and hence could not be presented as mean ± SD. ^#^ NEB-Susp parameters are reproduced from our previous reported work [3]. ^d^ AUC and MRT in plasma are presented as mean ± SD of four measurements.

## Data Availability

The original contributions presented in the study are included in the article/Supplementary Materials; further inquiries can be directed to the corresponding author.

## Supplementary Materials

The following supporting information can be downloaded at: https://www.mdpi.com/article/10.3390/nano14161347/s1, Figure S1: Particle size and distribution of the optimized NEB-PNPs analyzed using Malvern Zeta-sizer. Figure S2: (A) DSC thermograms of (i) NEB, (ii) PCL, (iii) physical mixture of NEB with various excipients used in the preparation of NEB-PNPs, (iv) freeze-dried powder of NEB-PNPs, and (v) trehalose; (B) The pXRD graphs of (i) NEB, (ii) PCL, (iii) physical mixture of NEB with various ingredients used in the formulation of NEB-PNPs, (iv) trehalose, and (v) freeze-dried NEB-PNPs. Figure S3: Linear plot of storage modulus (G’, Pa) versus temperature of blank ISG and NEB-PNPs-ISG. Note: A—blank ISG; B—NEB-PNPs-ISG; and C—NEB-PNPs-ISG in the presence of STF. Figure S4: Results obtained from stability studies of (a) freeze-dried powder of NEB-PNPs stored at 25 ± 2 °C and 60 ± 5% RH and (b) NEB-PNPs-ISG stored at 2–8 °C studied for 60-day period. Figure S5: Plasma NEB concentration versus time profiles of NEB-PNPs-Susp, NEB-PNPs-ISG, and NEB-Susp administered through ocular route. Note: Data of NEB-Susp are reproduced from our previous reported work [3]. Table S1: Factors and their levels/constraints used in BBD for optimization of NEB-PNPs. Table S2: BBD design matrix with levels of the three factors used in each experimental run in the preparation of NEB-PNPs and the corresponding observed values obtained for PS, DL (%), EE (%), and ZP.
