# Supplementary material for: Nebivolol Polymeric Nanoparticles-Loaded In Situ Gel for Effective Treatment of Glaucoma: Optimization, Physicochemical Characterization, and Pharmacokinetic and Pharmacodynamic Evaluation
Source: Nanomaterials (Basel). 2024 Aug 14;14(16):1347. doi: 10.3390/nano14161347 (PMC11356797; doi:10.3390/nano14161347)
Supplement: Supplementary file 1 [file nanomaterials-14-01347-s001.zip › nanomaterials-3137282-supplementary.pdf]

Nebivolol Polymeric Nanoparticles-Loaded In Situ Gel for Effective Treatment of Glaucoma: Optimization, Physicochemical Characterization, and Pharmacokinetic and Pharmacodynamic Evaluation

Pradeep Singh Rawat [1], Punna Rao Ravi [1,*], Mohammed Shareef Khan [1], Radhika Rajiv Mahajan [1] and Łukasz Szeleszczuk [2]

[1] Department of Pharmacy, Birla Institute of Technology and Science, Pilani Hyderabad Campus, Jawahar Nagar, Kapra Mandal, Medchal District, Hyderabad 500078, Telangana, India; p20170300@hyderabad.bits-pilani.ac.in (P.S.R.); p20190064@hyderabad.bits-pilani.ac.in (M.S.K.); p20200469@hyderabad.bits-pilani.ac.in (R.R.M.)

[2] Department of Organic and Physical Chemistry, Faculty of Pharmacy, Medical University of Warsaw, Banacha 1 Str., 02-093 Warsaw, Poland; lukasz.szeleszczuk@wum.edu.pl

* Correspondence: rpunnarao@hyderabad.bits-pilani.ac.in; Tel.: +91-4066303539 or +91-7730910268

SUPPLEMENTARY DATA

SUPPLEMENTARY FIGURES

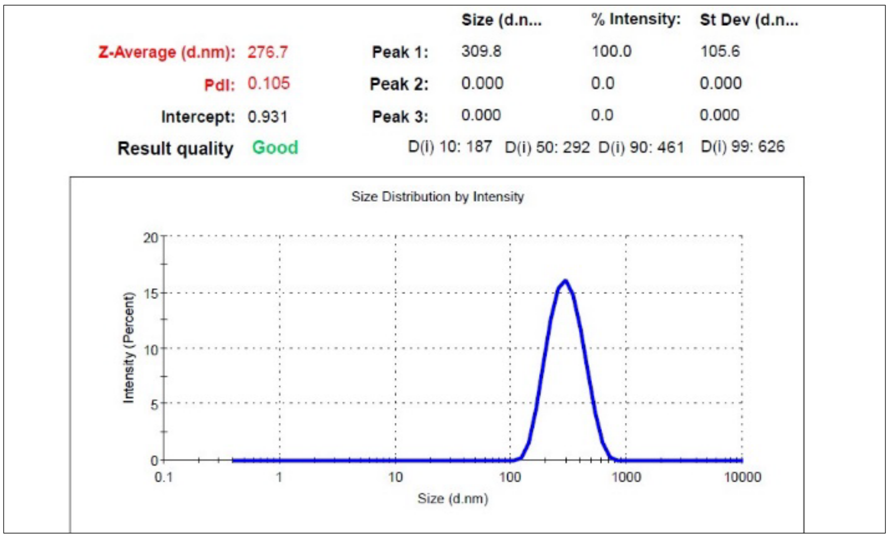

Figure S1: Particle size and distribution of the optimized NEB-PNPs anlaysed using Malvern Zeta-sizer.

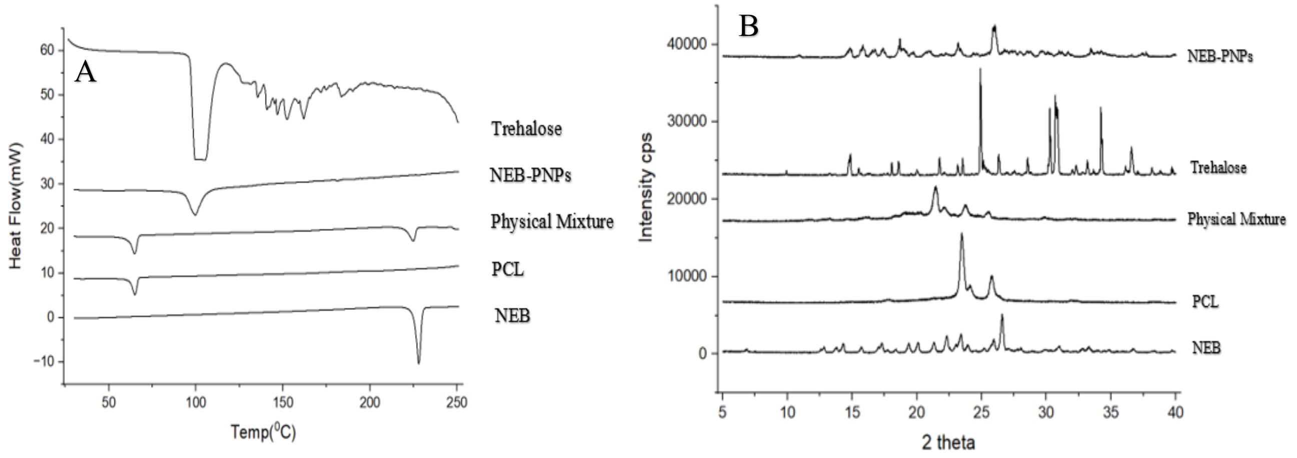

Figure S2: A) DSC thermograms of (i) NEB, (ii) PCL (iii) Physical mixture of NEB with various excipients used in the preparation of NEB-PNPs (iv) freeze-dried powder of NEB-PNPs and (v) Trehalose. B) The pXRD graphs of (i) NEB, (ii) PCL, (iii) Physical mixture of NEB with various ingredients used in the formulation of NEB-PNPs, (iv) Trehalose and (v) freeze-dried NEB-PNPs.

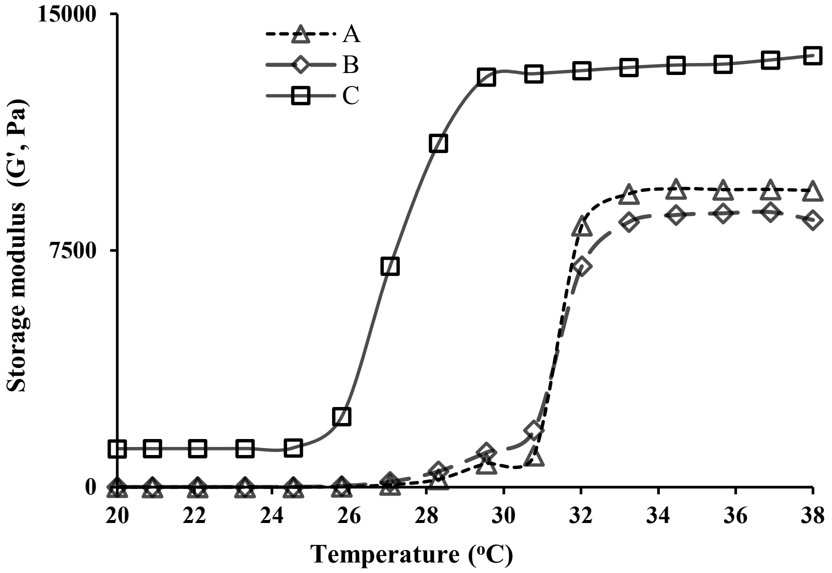

Figure S3: Linear plot of storage modulus (G', Pa) versus temperature of blank ISG and NEB-PNPs-ISG. Note: A- blank ISG; B- NEB-PNPs-ISG and C- NEB-PNPs-ISG in the presence of STF.

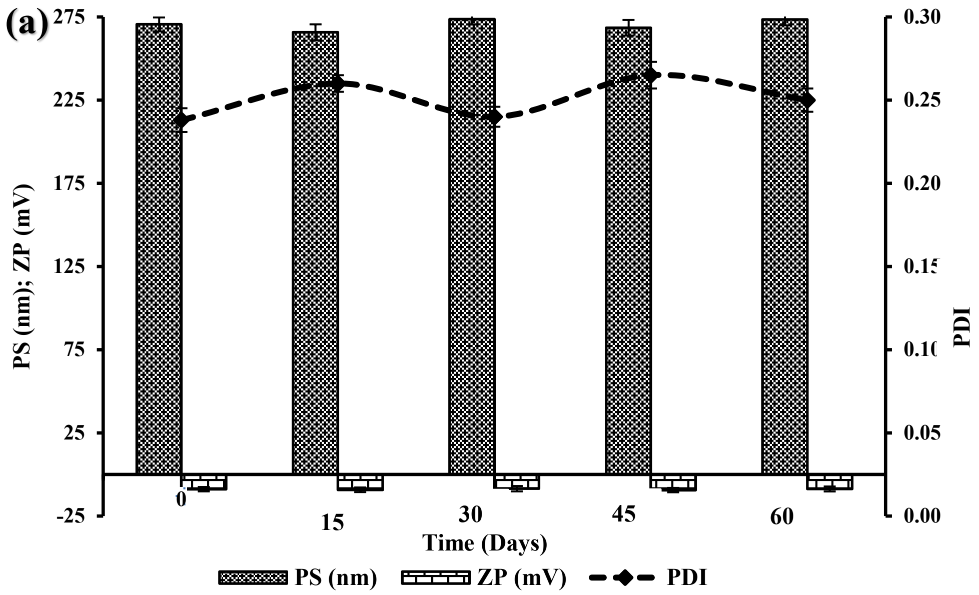

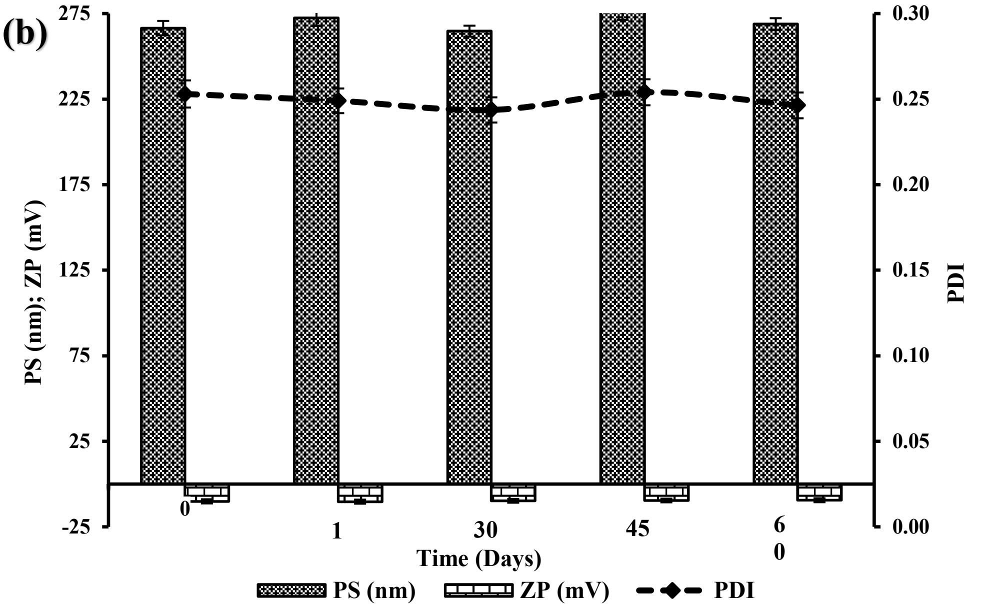

Figure S4: Results obtained from stability studies of (a) freeze dried powder of NEB-PNPs stored at 25±2 °C and 60±5% RH and (b) NEB-PNPs-ISG stored at 2-8 °C studied for 60-day period.

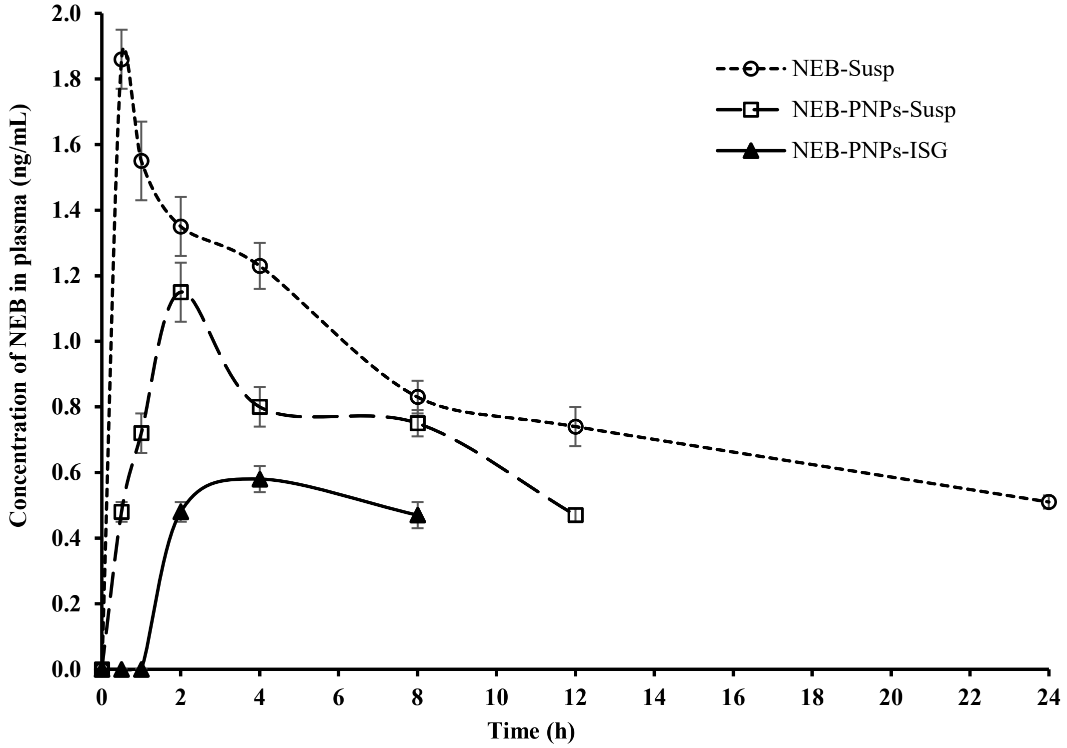

Figure S5: Plasma NEB concentration versus time profiles of NEB-PNPs-Susp, NEB-PNPs-ISG and NEB-Susp administered through ocular route. Note: Data of NEB-Susp is reproduced from our previous reported work [3]..

SUPPLEMENTARY TABLES

Table S1 Factors and their levels/constraints used in BBD for optimization of NEB-PNPs.

Factors	Levels used		
Independent variables	-1	0	+1
X_1 = Amount of PCL (mg)	20	45	70
X_2 = Concentration of PVA (% w/v)	0.5	0.75	1.0
X_3 = Homogenization speed (rpm)	5000	7500	10000
Dependent variables	Constraints		
Y_1 = PS (nm)	Minimize		
Y_2 = DL (%)	Maximize		

Table S2 BBD design matrix with levels of the three factors used in each experimental run in the preparation of NEB-PNPs and the corresponding observed values obtained for PS, DL (%), EE (%) and ZP.

Run No.	Amount of Polycaprolactone (X_1) (mg)	Concentration of PVA (X_2) (% w/v)	Homo. Speed (rpm)	PS (Y_1) (nm)	DL (Y_2) (%)	EE (%)	ZP (mV)
1	55	0.5	5000	344.7	14.8	96.6	-6.4
2	55	0.75	7500	303.9	14.8	96.5	-6.2
3	100	0.75	10000	320.0	8.7	96.7	-6.4
4	55	1	10000	299.5	14.7	96.5	-6.0
5	10	1	7500	256.8	47.1	96.6	-8.6
6	55	1	5000	310.5	14.7	96.7	-6.6
7	100	0.5	7500	381	8.7	96.9	-5.6
8	10	0.75	10000	240.6	48.0	96.6	-8.8
9	55	0.75	7500	291.7	14.7	96.5	-8.2
10	55	0.75	7500	271	14.8	96.5	-8.7
11	100	0.75	5000	437.6	8.6	96.8	-4.7
12	55	0.5	10000	268.7	14.7	96.6	-8.3
13	10	0.5	7500	259.3	47.8	97.0	-8.4
14	55	0.75	7500	277.7	14.8	96.7	-8.8
15	55	0.75	7500	287.2	14.8	96.5	-8.2
16	10	0.75	5000	296.9	47.8	96.9	-8.5
17	100	1	7500	380.6	8.7	96.8	-5.5

Note: The response data are shown as the average of three independent measurements with %RSD of less than 3% for the three measurements.
